# Berberine Alleviates Olanzapine-Induced Adipogenesis via the AMPKα–SREBP Pathway in 3T3-L1 Cells

**DOI:** 10.3390/ijms17111865

**Published:** 2016-11-09

**Authors:** Yanjie Li, Xiaomin Zhao, Xiyu Feng, Xuemei Liu, Chao Deng, Chang-Hua Hu

**Affiliations:** 1 School of Pharmaceutical Sciences, Southwest University, Chongqing 400715, China; anlan8934@email.swu.edu.cn (Y.L.); zxmqingting1219@163.com (X.Z.); fengxiyu1991858@163.com (X.F.); liuxm@swu.edu.cn (X.L.); 2 Engineer Research Center of Chongqing Pharmaceutical Process and Quality Control, Chongqing 400715, China; 3 Antipsychotic Research Laboratory, School of Medicine, and Illawarra Health and Medical Research Institute, University of Wollongong, Wollongong 2522, NSW, Australia; chao@uow.edu.au

**Keywords:** berberine, olanzapine, adipogenesis, AMPKα, SREBPs, 3T3-L1 cells

## Abstract

The aim of this study was to investigate the mechanisms underlying the inhibitory effects of berberine (BBR) on olanzapine (OLZ)-induced adipogenesis in a well-replicated 3T3-L1 cell model. Oil-Red-O (ORO) staining showed that BBR significantly decreased OLZ-induced adipogenesis. Co-treatment with OLZ and BBR decreased the accumulation of triglyceride (TG) and total cholesterol (TC) by 55.58% ± 3.65% and 49.84% ± 8.31%, respectively, in 3T3-L1 adipocytes accompanied by reduced expression of Sterol regulatory element binding proteins 1 (SREBP1), fatty acid synthase (FAS), peroxisome proliferator activated receptor-γ (PPARγ), SREBP2, low-density lipoprotein receptor (LDLR), and hydroxymethylglutaryl-coenzyme A reductase (HMGR) genes compared with OLZ alone. Consistently, the co-treatment downregulated protein levels of SREBP1, SREBP2, and LDLR by 57.71% ± 9.42%, 73.05% ± 11.82%, and 59.46% ± 9.91%, respectively. In addition, co-treatment reversed the phosphorylation level of AMP-activated protein kinase-α (AMPKα), which was reduced by OLZ, determined via the ratio of pAMPKα:AMPKα (94.1%) compared with OLZ alone. The results showed that BBR may prevent lipid metabolism disorders caused by OLZ by reversing the degree of SREBP pathway upregulated and the phosphorylation of AMPKα downregulated. Collectively, these results indicated that BBR could be used as a potential adjuvant to prevent dyslipidemia and obesity caused by the use of second-generation antipsychotic medication.

## 1. Introduction

Second-generation antipsychotic drugs (SGAs), such as olanzapine (OLZ), have become first line medication for treating schizophrenia symptoms and other mental disorders due to their lower incidence of extra pyramidal symptoms (EPS) and improved tolerability compared with first-generation antipsychotic drugs (FGAs) [1,2]. However, they cause troublesome metabolic side-effects such as SGA-jnduced metabolic abnormalities, increased leptin [3], and decreased adiponectin levels [4], weight gain, dyslipidemia, insulin resistance, and type II diabetes mellitus, which could further lead to the risk of cardiovascular disease and premature death [5,6,7,8].

In past decades, accumulating evidence has indicated that OLZ could upregulate the transcriptional level of sterol regulatory element binding protein (SREBP), which is a key factor for modulating lipid homeostasis in cultured cells (including glioma cells, liver cells, and adipocytes) and rats [9,10,11,12,13]. The SREBP family has three isoforms—SREBP-1a, SREBP-1c and SREBP-2—that are involved in lipogenesis and cholesterogenesis [14,15,16,17]. Furthermore, it has been demonstrated that OLZ could augment SREBP-dependent lipid synthesis by suppressing AMP-activated protein kinase α (AMPKα) activity in hepatocytes [10]. AMPKα is a major transcriptional regulator for controlling hepatic energy metabolism [18,19,20]. It is plausible to suspect that AMPKα activation would significantly ameliorate lipid metabolism dysregulation that occurs during OLZ treatment.

BBR, an isoquinoline derivative alkaloid extracted from Berberis and Coptis herbs such as *Hydrastis canadensis* (goldenseal), *Cortex phellodendri* (Huangbai), and *Rhizoma coptidis* (Huanglian), is a commonly used traditional Chinese medicine for treatment of microbial diarrhea [21,22]. In addition, BBR has potential pharmacological effects, including effects on metabolic abnormalities, and antiarrhythmic, antihypertensive, anticancer, antidiabetic, and antihyperlipidenmic effects [23,24,25,26]. Accumulated evidence has shown that BBR can reduce lipogenesis in vitro and in vivo through a series of mechanisms, including the downregulation of the expression of peroxisome proliferator activated receptor γ (PPARγ) [27]. Although BBR has been reported to inhibit clozapine and risperidone-induced adipogenesis via SREBP-1 in 3T3-L1 cells [28], it is unclear whether SREBP-2 plays a role in the BBR restraint of OLZ-induced adipogenesis. Likewise, BBR ameliorates lipid dysregulation in obesity by managing the activity of peripheral and central AMPKα [29]. Additionally, it has been shown that AMPK directly inhibits expression and/or activity of SREBP-1c [19,30]. Therefore, BBR has the potential to prevent OLZ-induced dyslipidemia by acting on these relevant pathways.

In this study, we established an OLZ-induced adipogenesis model in 3T3-L1 adipocytes, which was used to investigate multiple key genes that are involved in adipogesis (SREBP1, fatty acid synthetase (FAS) and PPARγ) and cholesterogenesis (SREBP2, low-density lipoprotein receptor (LDLR) and hydroxymethylglutaryl coenzyme A reductase (HMGR)). Furthermore, we examined whether BBR modulates gene transcription and protein expression levels of the AMPKα–SREBP pathway and its role in downregulating OLZ-induced adipogenesis in a 3T3-L1 cell model.

## 2. Results

### 2.1. Effects of Treatment with OLZ, BBR, or Both on 3T3-L1 Differentiation

**Figure 1:**
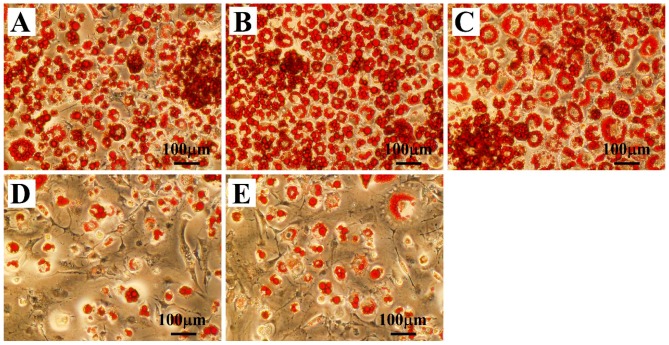
Olanzipine (OLZ) + Berberine (BBR) co-treatment reduced lipid droplet accumulation induced by OLZ in 3T3-L1 adipocytes. Representative images were randomly selected and sections of 3T3-L1 adipocytes stained with Oil-Red-O (ORO) treated with drugs. (**A**–**E**) ORO-stained cell morphology of lipid droplets in 3T3-L1 adipocytes treated with Vehicle (**A**); 1 μM Rosiglitazone (Ros) (**B**); OLZ alone (**C**); BBR alone (**D**); and OLZ + BBR co-treatment (**E**). Treated with DMSO as control. Treatment of 1 μM Ros used as positive control. Scale bars, 100 μm.

To detect whether OLZ could increase the rate of adipogenesis, 3T3-L1 cells were exposed to OLZ (0, 1, 10, and 50 μM) with DM for the first six days (Days 0–6), and were then stained with Oil-Red-O (ORO) on Day 12. The effect of OLZ in facilitating differentiation of 3T3-L1 cells had a dose-dependent response (Supplemental Experimental Section 1: Effects of Treatment with OLZ on 3T3-L1 Differentiation). 3T3-L1 adipocytes treated with 10 μM OLZ tended to be much larger and prone to rupture, suggesting this concentration of OLZ could have the greatest effect on adipogenesis (Figure S1). On this basis, 10 μM OLZ was used in the following investigation of BBR’s inhibitory effects. Additionally, various concentrations of BBR (0, 0.675, 1.25, 2.5, and 5 μM) were added to the 3T3-L1 cells culture for six days (Supplemental Experimental Section 2: Effects of Treatment with BBR on 3T3-L1 Differentiation). The inhibitory effect of BBR on adipogenesis of 3T3-L1 cells was a dose-dependent response, and 5 μM BBR clearly reduced the number of lipid droplets stained with ORO (Figure S2). Therefore, 5 μM BBR combined with 10 μM OLZ were added to the DM for six days during the 3T3-L1 cell culture. Microscopic images of ORO staining showed that 5 μM BBR could significantly reverse the enhancement of OLZ-induced oil droplet accumulation (Figure 1A–E).

### 2.2. Effects of Treatment with OLZ, BBR, or Both on Biochemical Properties of 3T3-L1 Cells

**Figure 2:**
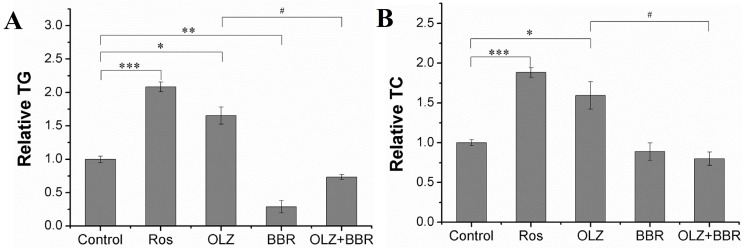
OLZ + BBR co-treatment could decrease the biochemical properties induced by OLZ in 3T3-L1 adipocytes. The determination of triglyceride (TG) (**A**) and total cholesterol (TC) (**B**) accumulation in 3T3-L1 adipocytes treated with OLZ, BBR, or both on Day 12. Treatment with DMSO as the control. Treatment of 1 μM Ros used as positive control. Values given are the mean ± SEM (*n* = 3). (* *p* < 0.05, ** *p* < 0.01 and *** *p* < 0.001 vs. control, ^#^ *p* < 0.05 vs. treated with OLZ alone).

A 10 μM dose of OLZ-induced accumulation of triglycerides (TG) compared with controls (*p* < 0.05). A 5 μM BBR dose significantly decreased the degree of TG accumulation compared with the control (71.05% ± 9.2%, *p* < 0.01, Figure 2), which was consistent with the reduction in adipogenesis shown in cell imaging (Figure S2). Moreover, in order to study the candidate inhibitory effects of BBR on OLZ-induced adipogenesis, 5 μM BBR and 10 μM OLZ were exposed to 3T3-L1 cells cultures. The results revealed that 3T3-L1 cells incubated in the presence of OLZ + BBR for six days decreased by 55.58% ± 3.65% compared with treatment with OLZ alone (*p* < 0.05) (Figure 2A). A potent PPARγ agonist and 1 μM Rosiglitazone (Ros) significantly increased adipogenesis and enhanced the degree of TG and total cholesterol (TC) accumulation approximately 2-fold and 1.8-fold (*p* < 0.001), respectively. In addition, the TC assay suggested that treatment with OLZ alone could significantly enhance TC accumulation (59.5% ± 17.35%, *p* < 0.05). OLZ + BBR co-treatment markedly reduced the accumulation of TC induced by OLZ in 3T3-L1 cells (49.84% ± 8.31%, *p* < 0.05) (Figure 2B). However, treatment with BBR alone had no observable effect on TC accumulation.

### 2.3. Effects of Treatment with OLZ, BBR, or Both on the Expression of SREBP-Related Genes for Adipogenesis in 3T3-L1 Cells

**Figure 3:**
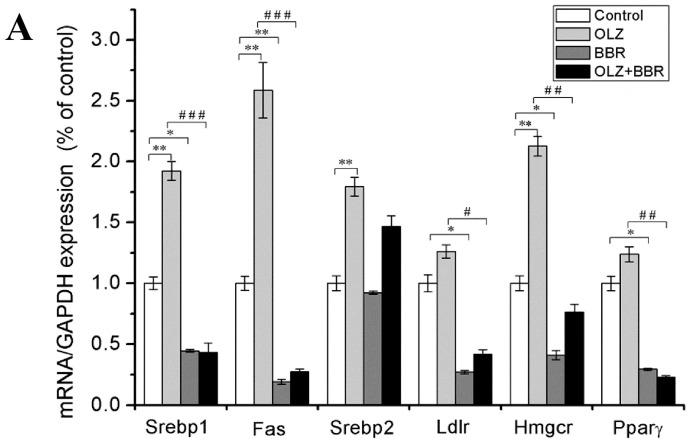

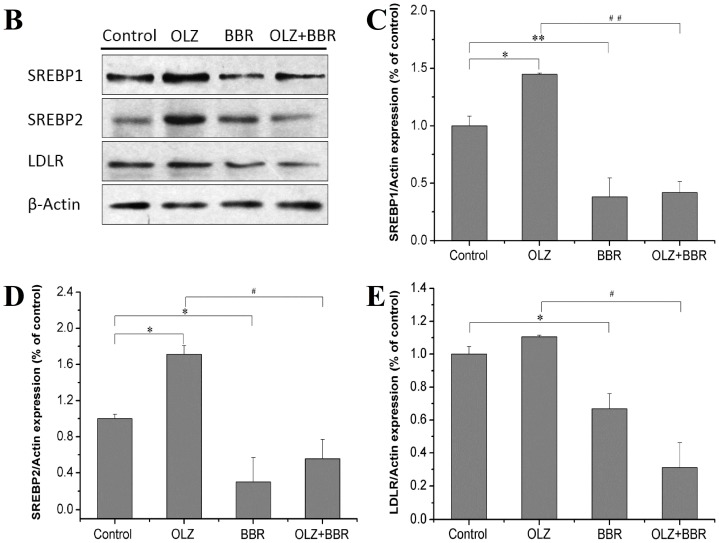
Effects of treatment with OLZ, BBR, or both on the expression of sterol regulatory element binding protein (SREBP) pathway in 3T3-L1 adipocytes. (**A**) The mRNA expression levels of Srebp1, fatty acid synthetase (Fas), SREBP2, low-density lipoprotein receptor (LDLR), hydroxymethylglutaryl-coenzyme A reductase (HMGR), and peroxisome proliferator activated receptor-γ (PPARγ) relative to GAPDH during 3T3-L1 cells treated with OLZ, BBR, or both for 72 h; (**B**) the protein expression levels of SREBP-1, SREBP-2, and LDLR relative to β-Actin of 3T3-L1 cells treated with OLZ, BBR, or both for 72 h and their quantification, respectively (**C**–**E**). Treatment with DMSO as control. Values given are the mean ± SEM (*n* = 3). * *p* < 0.05, ** *p* < 0.01 vs. control, ^#^ *p* < 0.05, ^##^ *p* < 0.01 and ^###^ *p* < 0.001 vs. treated with OLZ alone.

Figure 3A shows the effects of treatment with OLZ, BBR, or both on mRNA expression of the SREBP pathway. Treatment with OLZ alone significantly augmented the mRNA expression of genes involved in adipogenesis including *Srebp1* and the downstream gene *Fas* compared with the control group (92.4% ± 7.76%, 158.75% ± 22.73%, both *p* < 0.01). Treatment with BBR alone clearly decreased the mRNA expression levels of Srebp1 and Fas compared with the control (55.45% ± 1.12%, *p* < 0.05, 80.79% ± 9.88%, *p* < 0.01, respectively). The OLZ + BBR co-treatment group markedly reversed the upregulation of *Srebp1* and *Fas* gene expression levels induced by OLZ alone (77.48% ± 7.66%, 81.37% ± 1.14%, both *p* < 0.001). Compared with the control, treatment with BBR alone clearly reduced the gene expression level of *Pparγ*, which was a member of the adipogenesis related transcription factors (70.51% ± 0.92%, *p* < 0.05). The OLZ + BBR co-treatment group distinctly lowered the mRNA expression levels of Pparγ compared with OLZ alone (65.05% ± 9.04%, *p* < 0.01). However, treatment with OLZ alone tended to have a higher expression of *Pparγ* gene expression levels than the control group (*p* = 0.07) (Figure 3A). Treatment with OLZ alone also significantly increased the protein expression level of SREB1 compared with the control (53.54% ± 8.96%, *p* < 0.01). Treatment with BBR alone decreased the protein expression of SREB1 compared with the control (43.43% ± 16.4%, *p* < 0.05). The OLZ + BBR co-treatment group showed observably lower SREB1 protein expression compared with OLZ alone (57.71% ± 9.42%, *p* < 0.01) (Figure 3B,C).

We also measured the gene expression levels of *Srebp2*, *Ldlr*, and *Hmgr*, which were involved in cholesterolopoiesis. The treatment with OLZ alone induced a significant elevation of Srebp2 and Hmgr mRNA expression compared with the control group (79.37% ± 7.67%, 112.82% ± 8.07%, both *p* < 0.01). However, treatment with OLZ alone tended to increase mRNA expression of Ldlr compared with the control (*p* = 0.06). Treatment with BBR alone group reduced Ldlr and Hmgr markedly compared with the control group (72.87% ± 11.36%, 59.06% ± 8.38%, both *p* < 0.05). Nonetheless, when treated with BBR alone, there was no significant augmentation in mRNA expression level of Srebp2 compared with the control (Figure 3A). Additionally, SREBP2 and LDLR protein expression levels were consistent with that of their gene expression. Compared with the control, treatment with OLZ alone significantly augmented the protein expression level of SREBP2 (94.27% ± 9.62%, *p* < 0.001) and LDLR (31.03% ± 0.94%, *p* < 0.05). On the other hand, treatment with BBR alone decreased the protein expression level of SREBP2 and LDLR compared with the controls (35.2% ± 10.49%, 28.47% ± 9.03%, both *p* < 0.05). OLZ + BBR co-treatment of 3T3-L1 cells distinctly decreased the protein expression level of SREBP2 and LDLR compared with the group treated with OLZ alone (73.05% ± 11.82%, *p* < 0.001, 59.46% ± 9.91%, *p* < 0.01) (Figure 3B,D,E).

### 2.4. Effects of Treatment with OLZ, BBR, or Both on Expression of AMPKα-Related Adipogenesis in 3T3-L1 Cells

**Figure 4:**
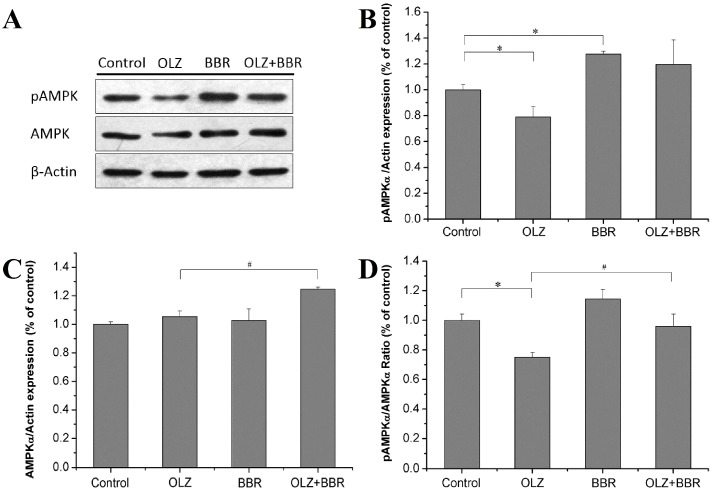
Effects of treatment with OLZ, BBR, or both on the expression of AMP-activated protein kinase-α (AMPKα) in 3T3-L1 adipocytes. (**A**) The protein expression levels of pAMPKα and AMPKα relative to β-Actin in 3T3-L1 cells treated with OLZ, BBR, or both for 72 h and their quantification, respectively (**B**–**D**). Treatment with DMSO as control. Values given are the mean ± SEM (*n* = 3). * *p* < 0.05 vs. control, ^#^ *p* < 0.05 vs. treated with OLZ alone.

There was a clearly attenuated effect on the protein expression level of pAMPKα in 3T3-L1 cells treated with OLZ alone compared with the control (22.51% ± 8%, *p* < 0.05) (Figure 4A,B). In comparison with the control, treatment with BBR alone had an obviously elevated protein expression of pAMPKα (20.7% ± 2.1%, *p* < 0.05). OLZ + BBR co-treatment of 3T3-L1 cells enhanced the protein expression of pAMPKα, but it was not significant (*p* = 0.08). Treatment with OLZ alone had a significant effect on the protein expression level of AMPKα compared with the control (43% ± 3.95%, *p* < 0.01). OLZ + BBR co-treatment group also decreased the protein expression level of AMPKα by 24.07% ± 1.47% compared with the treatment with OLZ alone (*p* < 0.05). No significant differences were observed in the protein expression level of AMPKα in treatment with BBR alone compared with the control (Figure 4A–C). Treatment with OLZ alone clearly decreased the ratio of pAMPKα:AMPKα compared with the control group (45.81% ± 3.22%, *p* < 0.05) (Figure 4D) whereas treatment with BBR alone increased the ratio of pAMPKα:AMPKα by 36.11% ± 6.35% compared with the control (*p* < 0.05). The OLZ + BBR co-treatment group increased the ratio of pAMPKα:AMPKα significantly compared with OLZ-only group (94.09% ± 17.81%, *p* < 0.01) (Figure 4D).

## 3. Discussion

In the present study, we investigated the inhibitory effect and underlying mechanisms of BBR on OLZ-induced adipogenesis. OLZ + BBR co-treatment clearly inhibited adipogenesis and reversed the enhancement of gene and protein expression levels of the SREBP pathway compared with treatment with OLZ alone. In addition, the OLZ + BBR co-treatment had a significant rebound effect on the phosphorylation level of AMPKα compared with the OLZ-only group. In summary, OLZ + BBR co-treatment could reduce adipogenesis by modulating the SREBPs-AMPKα pathway.

Our data demonstrated that 10 μM OLZ could significantly augment the adipogenesis. A 10 μM dose of OLZ increased mRNA expression levels of Srebp-related genes and improved the protein expression levels of SREBP1, SREBP2, and LDLR. These results showed that OLZ upregulation of the SREBP pathway was in relation to adipogenesis, which was consistent with the reports on several cell lines and primary rat hepatocytes [11,12,31]. However, the ascending trend of mRNA expression of Srebp2 was not significant, so it might be associated with emerging feedback adjustments in lipid metabolism [32,33,34]. Simultaneously, we found that OLZ restrained the phosphorylation level of pAMPKα in 3T3-L1 cells. AMPKα, a well-known metabolic regulator of energy metabolism, always plays an important role at the cellular, as well as the whole body, level. There is some evidence that AMPKα phosphorylation is facilitated in the hypothalamus and reduced in hepatocytes by OLZ, implying that central and peripheral AMPKα activities could be dissimilarly regulated by the identical stimulus [10,35,36,37]. Additionally, several studies have reported that AMPKα reduced lipid synthesis by restraining SREBP activity and led to fatty acid oxidation in the liver to control hepatic energy metabolism [18,19,20,38]. Like the effects of OLZ on hepatic AMPKα, our results suggested that AMPKα activity was inhibited and that the SREBP pathway was upregulated in adipocytes upon exposure to OLZ. Hence, our results showed that OLZ inhibited the phosphorylation of AMPKα to increase the SREBP-regulated adipogenesis in 3T3-L1 cells.

Recently, it was reported that BBR could inhibit adipogenesis induced by clozapine and risperidone through the SREBP-1-related pathway in 3T3-L1 cells [28]. BBR can also prevent weight gain induced by OLZ via the UCP-1-related pathway in rats [39]. In addition, pharmaceutical compositions containing BBR could also be used to treat or prevent weight gain and obesity associated with anti-psychotic drugs [40]. These reports indicate the effectiveness of BBR as an add-on treatment in subjects starting treatment with OLZ for controlling SGA-related metabolic disorders. Our data suggests that BBR had inhibitory effects on lipid metabolism by reducing the mRNA expression as well as directly decreasing the protein expression of SREBP1, SREBP2, and LDLR. The inhibitory effect involved in the SREBP-1 pathway was consistent with a previously reported impact of BBR on adipogenesis induced by the clozapine and risperidone in 3T3-L1 cells [28]. However, the underlying mechanisms of the inhibitory effect of BBR on cholesterolopoiesis were not entirely clear, and further investigation is needed. In this study, BBR alone was able to downregulate the expression of SREBP2 in relation to cholesterolopoiesis. Recently, it was determined that BBR also exerted inhibitory actions on adipogenesis by improving the activation of AMPKα and promoting adiponectin multimerization in 3T3-L1 cells [41]. In this study, BBR alone inhibited AMPKα–SREBP-related adipogenesis in 3T3-L1 adipocytes. It has recently been reported that betahistine and OLZ co-treatment ameliorated OLZ-induced weight gain and dyslipidemia through the modulation of histaminergic, AMPKα–SREBP1, and PPARγ-dependent pathways [42]. Our data provides preliminary evidence that BBR + OLZ co-treatment can prevent adipogenesis induced by OLZ. 

It is noteworthy that there are several limitations in this study. Although this study showed that BBR reduced the differentiation of adipocytes, cell division or the apoptosis of adipocytes were not examined. Therefore, we could not completely exclude the possible effects of BBR on cell division or the apoptosis of adipocytes, which could be an important issue for future studies. Another limitation is that the current study has been conducted only in a cell culture; further study is necessary to investigate whether BBR has the same inhibitory effect on dyslipidemia induced by OLZ in an animal model for schizophrenia, which may provide crucial information for potential clinical trials to control dyslipidemia caused by OLZ and other SGAs in patients with schizophrenia.

## 4. Materials and Methods

### 4.1. Materials

3T3-L1 cells were purchased from the Institute of Basic Medical Sciences, Chinese Academy of Medical Sciences Cell Resource Center (Beijing, China). Berberine was purchased from the National Institutes for Food and Drug Control (Beijing, China). An RNA Simple Total RNA Kit was purchased from Tiangen Biotech Co., Ltd. (Beijing, China), a Transcriptor First Strand DNA Synthesis Kit was obtained from Roche Diagnostics GmbH. (Basel, Switzerland). A SYBR Select Master Mix was purchased from Thermo Fisher Scientific, Inc. (Foster City, CA, USA), and a Protein Assay Kit was purchased from Transgen Biotech Co., Ltd. (Beijing, China). Commercially available TG and TC assay kits were purchased from Nanjing Jiancheng Bioengineering Institute (Nanjing, China). Primary antibodies against SREBP1, SREBP2, and LDLR were obtained from Abcam, Inc. (Cambridge, MA, USA), and β-Actin was obtained from Beijing Bioss Biotech Co., Ltd. (Beijing, China). Primary antibodies against AMPKα, pAMPKα, and anti-rabbit IgG-conjugated horseradish peroxidase (HRP) antibody were purchased from Cell Signaling Technology, Inc. (Beverly, MA, USA).

### 4.2. Culturing 3T3-L1 Fibroblasts and Differentiation into Adipocytes

**Figure 5:**
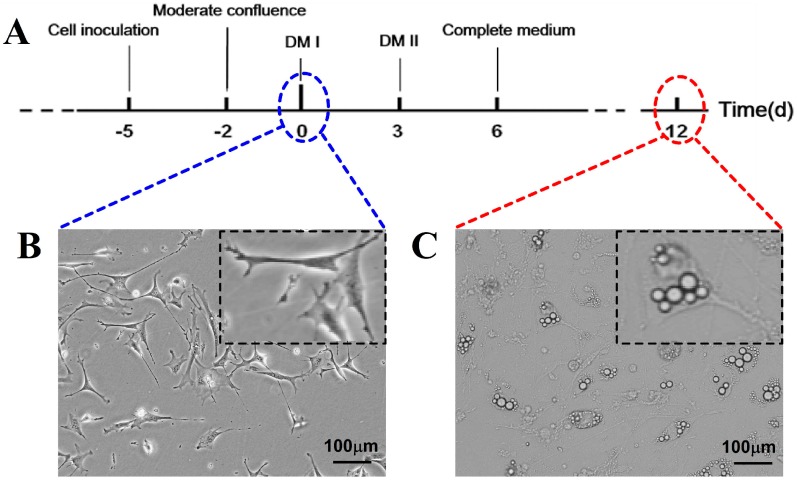
The condition of 3T3-L1 fibroblasts differentiation into adipocytes. (**A**) The differentiation protocol of 3T3-L1 into adipocytes; (**B**) the microscopic image of 3T3-L1 fibroblasts; (**C**) the microscopic image of 3T3-L1 adipocytes. DM: Abbreviation of differentiation medium. Scale bars, 100 μm. Inset images were magnified to highlight the cell morphology.

3T3-L1 fibroblasts were grown and maintained in DMEM containing 10% fetal bovine serum (FBS) (Figure 5A). To guarantee better observation clarity, differentiation began 2 days after the cells reached moderate confluence (denoted as Day 0) (Figure 5B), and cells were fed with prior-differentiation medium (DM) (DMEM, 10% FBS, 2 μg/mL of insulin, 100 ng/mL of dexamethasone, 500 μM 3-isobutyl-1-methylxanthine (IBMX), 100 ng/mL of D-Biotin). On Day 3, cells were incubated with post-differentiation medium (DMEM, 10% FBS, 2 μg/mL Insulin) for a further 3 days. Thereafter, adipocytes were fed with complete medium (DMEM, 10% FBS) every 3 days. A small amount of old medium was be removed gently. Cells were ready for use on Day 12 (Figure 5C). Lipid droplets formed by TG accumulation were clearly presented in adipocytes.

### 4.3. Oil-Red-O Staining

Differentiated 3T3-L1 cells were washed twice with PBS on Day 12. Monolayer cells were fixed on culture dishes with 4% paraformaldehyde at 37 °C for 40 min. After being washed with chilled PBS for three times, cells were incubated with ORO solution (0.3% ORO, 60% isopropanol, and 40% water) for 2 h at 37 °C. They were then rinsed thoroughly with water to eliminate unbound dyestuff on the surface of the monolayer at least 4 times. The number of staining lipid droplets of adipocyte was observed and imaged under an inverted microscope.

### 4.4. TG and TC Assay

Differentiated 3T3-L1 cells were washed with cold PBS 3 times on Day 12 and then scraped, followed by centrifugation at 1000× *g* for 10 min. After the supernatant was discarded, the remaining pellets were combined with 300 μL of ice-cold PBS (pH 7.4). Cells were cleaved by sonication (power, 300 W; per 3–5 s; pause 25 s; repeat 3 to 5 times). The supernatant was collected after centrifugation at 12,000× *g* for 15 min, and the concentration was determined via Protein Assay Kit. The concentrations of TG and TC in supernatant were analyzed by enzymology using the kits. All experimental assays were performed in accordance with the manufacturer’s instructions.

### 4.5. Quantitative Real-Time PCR (qRT-PCR)

Total RNA isolation was obtained according to the protocol recommended by the manufacturers. Total RNA was dissolved in pure water and sample integrity was checked by 1.5% agarose gel electrophoresis and quantified at 260/280 nm. cDNA was synthesized using Transcriptor First Strand DNA Synthesis Kit. For qRT-PCR, reactions were performed on the MyCyclerTM system (Bio-Rad, Hercules, CA, USA) using the SYBGR Select Master Mix (Applied Biosystems, Foster City, CA, USA). The primer sequences are shown in Table 1. The relative mRNA levels of these genes were calculated by the 2^−ΔΔ*C*t^ method and normalized with control-treated groups.

### 4.6. Western Blots

3T3-L1 cells were washed with 4 °C PBS twice. A mixture of RIPA Lysates and 1% 0.5 mM PMSF was added to the monolayer cells on the ice. After the cells were gently shaken and beaten, the lysate was collected and immediately centrifuged at 12,000× *g* at 4 °C for 10 min. The supernatant was saved as a protein extraction at −86 °C.

Protein concentrations were quantified utilizing a Protein Assay Kit. The homogenized samples (30 μg) were separated by SDS-PAGE (sodium dodecyl sulfate-polyacrylamide gel electrophoresis) using the BioRad Electrophoretic System (80 V for 0.5 h followed by 120 V for 2 h) and then transferred to a PVDF membrane at 4 °C. The PVDF membrane was blocked with 5% non-fat milk in TBST (0.1% Tween 20 in Tris-buffered saline) at room temperature for 1 h. The membrane was washed with TBST for three times (per 10 min), followed by incubation with a primary antibody at 4 °C overnight. The membrane was rinsed three times with TBST, then incubated with horseradish peroxidase-conjugated (HRP) secondary antibody at room temperature for 1 h. The protein expression of SREBP1, SREBP2, LDLR, pAMPKα, AMPKα, and the loading control β-Actin was detected by the ECL kit standard protocol. The band intensities were analyzed and quantified using Quantity One software (Bio-Rad).

### 4.7. Statistics

Data analysis was performed by the SPSS 19.0 procedure (Chicago, IL, USA). Results were presented as the mean ± SEM (*x* ± *s*). Statistical analysis used by a one-way analysis of variance (ANOVA) followed by a Tukey’s post hoc test for multiple comparisons. *p* < 0.05 was considered statistically significant.

## 5. Conclusions

Our data demonstrated that OLZ could induce dyslipidemia side effects by the regulation of AMPKα–SREBP pathway. In addition, co-treatment with BBR could partially reduce OLZ-induced adipogenesis through the AMPKα–SREBP-related pathway in 3T3-L1 adipocytes. Nevertheless, we measured the biochemical criteria (TG and TC) and gene expression through qRT-PCR array analysis and Western blot in vitro, and it is necessary to further investigate in vivo. The results not only supply one possible mechanism of the decrease in OLZ-induced adipogenesis by BBR but also provide a combination therapy project for schizophrenia-induced dyslipidemia.

## Figures and Tables

**Table 1:** qRT-PCR primers.

Oligonucleotide	Sequences (5’—3’)
GAPDH-Forward	GGTGAAGGTCGGTGTGAACG
GAPDH-Reverse	CTCGCTCCTGGAAGATGGTG
SREBP1-Forward	ACAAAAGCAAATCACTGAAGGACC
SREBP1-Reverse	CGGGCTCAGAGTCACTACCACC
FAS-Forward	GCACTGACTGTCTGTTTTCCAA
FAS-Reverse	AGCATCAAGAGCAGCATTTTTA
SREBP2-Forward	ACAACACTGACCAGCACCCATAC
SREBP2-Reverse	AAGACGCTCAAGACAATCACACC
LDLR-Forward	GGGTTGATTCCAAACTCCACTCTA
LDLR-Reverse	ACTGAAAATGGCTTCGTTTATGAC
HmgcR-Forward	CCAAACCCCGTAACCCAAAG
HmgcR-Reverse	GATAAAACTGCCAGAGAGAAACACT
PPARγ-Forward	ACAGGAAAGACAACGGACAAATCA
PPARγ-Reverse	CTTCTACGGATCGAAACTGGCAC

## Supplementary Materials

Supplementary materials can be found at www.mdpi.com/1422-0067/17/11/1865/s1.

## Abbreviations

FGAs	first-generation antipsychotic drugs
SGAs	second-generation antipsychotic drugs
CNS	central nervous system
CREB	cAMP-response element-binding protein
C/EBPα	CCAAT/enhancer binding protein-α
SREBPs	sterol regulatory element binding proteins
LDLR	low-density lipoprotein receptor
FAS	fatty acid synthase
PPARγ	peroxisome proliferator activated receptor-γ
HMGR	hydroxymethylglutaryl-coenzyme A reductase
AMPKα	AMP-activated protein kinase-α
OLZ	olanzapine
BBR	berberine
Ros	rosiglitazone
TG	triglycerides
TC	total cholesterol
ORO	Oil-Red-O
