# Supplementary material for: Berberine Alleviates Olanzapine-Induced Adipogenesis via the AMPKα–SREBP Pathway in 3T3-L1 Cells
Source: Int J Mol Sci. 2016 Nov 9;17(11):1865. doi: 10.3390/ijms17111865 (PMC5133865; doi:10.3390/ijms17111865)
Supplement: Supplementary file 1 [file ijms-17-01865-s001.pdf]

Supplementary Materials: Berberine Alleviates Olanzapine-Induced Adipogenesis via the AMPKα–SREBP Pathway in 3T3-L1 Cells

Yanjie Li, Xiaomin Zhao, Xiyu Feng, Xuemei Liu, Chao Deng and Chang-Hua Hu

1. Effects of Treatment with OLZ on 3T3-L1 Differentiation

To detect whether OLZ could enhance adipogenesis, 3T3-L1 cells were exposed to OLZ (0, 1, 10, 50 μM) for the first six days of differentiation (Day 0–6). On day 12, cells were stained with ORO (Figure S1A). Results of the microscopic imaging and degree of TG suggested that a concentration of 10 μM OLZ could greatest extent augment adipogenesis compared with vehicle control (* $p < 0.05$), but not 50 μM OLZ. 1 μM Ros which is a potent PPARγ agonist significantly increased lipogenesis and enhanced about 2-fold TG accumulation (** $p < 0.01$) (Figure S1B).

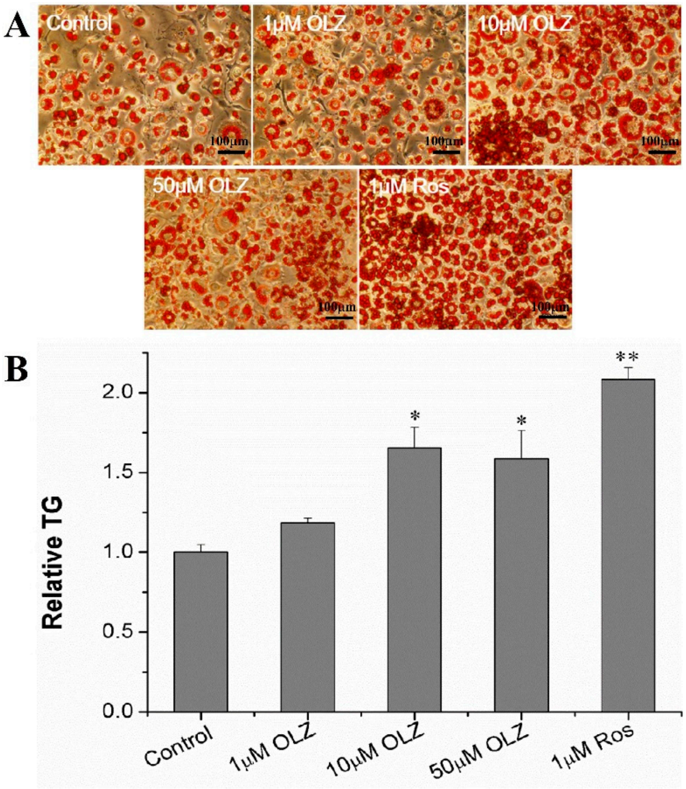

Figure S1. The effect of OLZ on 3T3-L1 differentiation. (**A**) Morphological change and oil droplet accumulation of 3T3-L1 cells differentiation treated with OLZ (0, 1, 10, 50μM); (**B**) the determination of TG in 3T3-L1 cells treated with OLZ on day 12. Treated with DMSO as vehicle control. Treatment 1 μM Ros as positive control group. The values given are the mean ± SEM ($n = 3$). (* $p < 0.05$ vs. vehicle control, ** $p < 0.01$ vs. vehicle control). Scale bars, 100 μm.

2. Effects of Treatment with BBR on 3T3-L1 Differentiation

To investigate the inhibitory effect of BBR on lipogenesis, various concentration BBR (0, 0.675, 1.25, 2.5, and 5 μM) were loaded to 3T3-L1 cells culture for 6 days. As shown in Figure S2A, the inhibitory effect of BBR on lipogenesis of 3T3-L1 cells was dose-dependent, 5 μM BBR could clearly reduce the shape of lipid droplets stained with Oil Red O. 5 μM BBR significantly decrease the degree of TG (28.95% ± 9.2%) compared to controls (** $p < 0.01$) (Figure S2B).

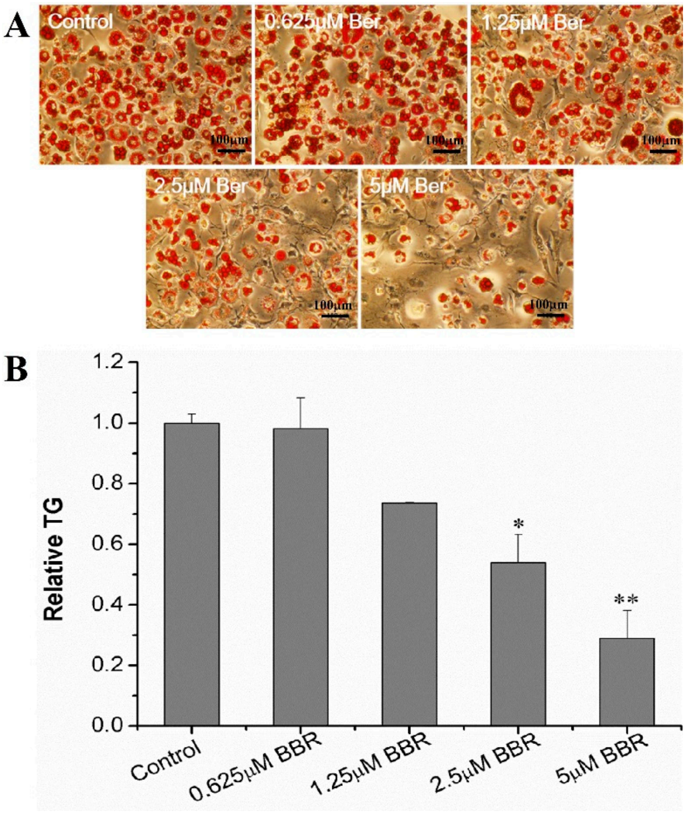

Figure S2. The effect of BBR on 3T3-L1 cells differentiation. (**A**) Cell morphology and (**B**) TG accumulation of 3T3-L1 adipocytes treated with BBR alone on day 12. The concentration of BBR respectively are 0, 0.625, 1.25, 2.5 and 5 μM. Treatment DMSO as control. Values given are the mean ± SEM ($n = 3$). (* $p < 0.05$ and ** $p < 0.01$ vs. control). Scale bars, 100 μm.
